# Supplementary material for: Splice-Junction-Based Mapping of Alternative Isoforms in the Human Proteome
Source: Cell Rep. Author manuscript; Available in PMC 2020 Jan 15. (PMC6961840; doi:10.1016/j.celrep.2019.11.026)
Supplement: 2 [file NIHMS1546469-supplement-2.zip › DF1/PXD006675/RightAtrium/RightAtrium_5_MYBPC3_RTDSHEDTGILDFSSLLK.pdf]

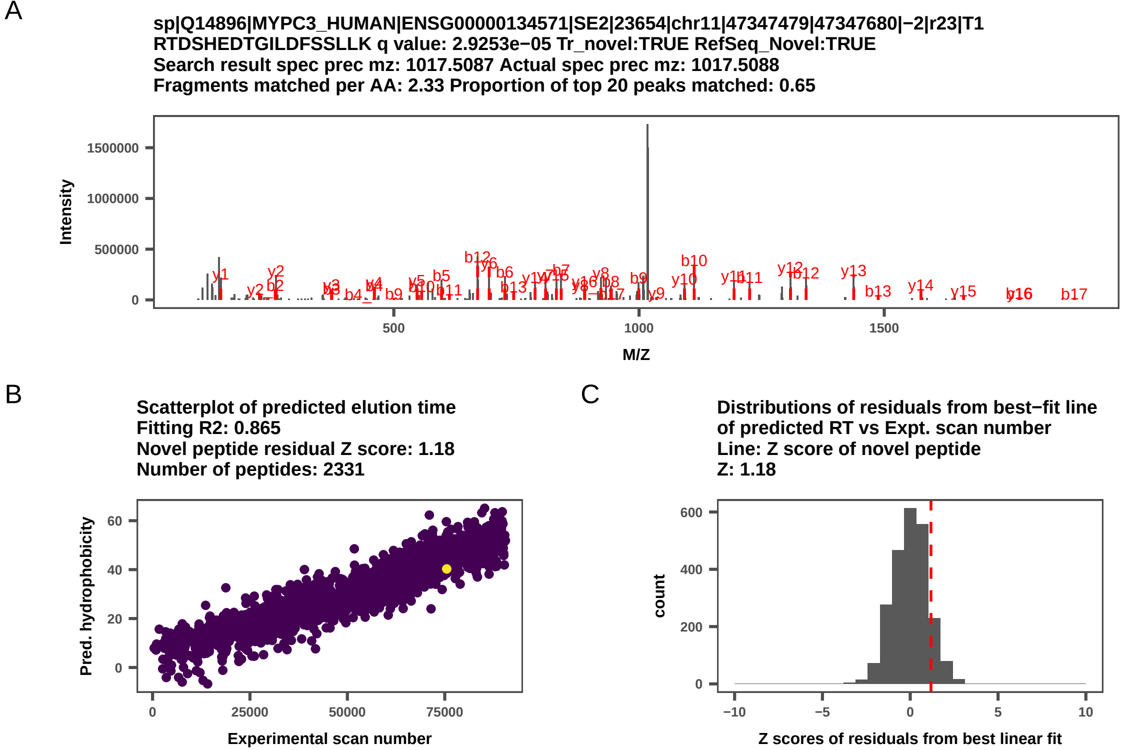

A
sp|Q14896|MYPC3_HUMAN|ENSG00000134571|SE2|23654|chr11|47347479|47347680|−2|r23|T1
RTDSHEDTGILDFSSLLK q value: 2.9253e−05 Tr_novel:TRUE RefSeq_Novel:TRUE
Search result spec prec mz: 1017.5087 Actual spec prec mz: 1017.5088
Fragments matched per AA: 2.33 Proportion of top 20 peaks matched: 0.65
Intensity
1500000
1000000
500000
0
500
1000
1500
M/Z
y1
y2
b2
y3
b4
y4
b9
y5
b5
b11
b12
y6
b6
b13
b7
y8
b8
b9
y9
b10
y10
b11
y12
b12
y13
b13
y14
y15
b16
b17
B
Scatterplot of predicted elution time
Fitting R2: 0.865
Novel peptide residual Z score: 1.18
Number of peptides: 2331
Pred. hydrophobicity
60
40
20
0
0
25000
50000
75000
Experimental scan number
C
Distributions of residuals from best−fit line
of predicted RT vs Expt. scan number
Line: Z score of novel peptide
Z: 1.18
count
600
400
200
0
−10
−5
0
5
10
Z scores of residuals from best linear fit
